# Train Repathing in Emergencies Based on Fuzzy Linear Programming

**DOI:** 10.1155/2014/598968

**Published:** 2014-07-10

**Authors:** Xuelei Meng, Bingmou Cui

**Affiliations:** School of Traffic and Transportation, Lanzhou Jiaotong University, P.O. Box 405, Anning West Road, Anning District, Lanzhou, Gansu 730070, China

## Abstract

Train pathing is a typical problem which is to assign the train trips on the sets of rail segments, such as rail tracks and links. This paper focuses on the train pathing problem, determining the paths of the train trips in emergencies. We analyze the influencing factors of train pathing, such as *transferring cost*, running cost, and social adverse effect cost. With the overall consideration of the segment and station capability constraints, we build the fuzzy linear programming model to solve the train pathing problem. We design the fuzzy membership function to describe the fuzzy coefficients. Furthermore, the contraction-expansion factors are introduced to contract or expand the value ranges of the fuzzy coefficients, coping with the uncertainty of the value range of the fuzzy coefficients. We propose a method based on triangular fuzzy coefficient and transfer the train pathing (fuzzy linear programming model) to a determinate linear model to solve the fuzzy linear programming problem. An emergency is supposed based on the real data of the Beijing-Shanghai Railway. The model in this paper was solved and the computation results prove the availability of the model and efficiency of the algorithm.

## 1. Introduction

Nowadays, railway transportation needs to become more and more competitive, so new features are required to improve the planning process. There are two approaches to improve the capacity of the railway infrastructure. One is to enhance the construction of railway infrastructure, such as extending the rail tracks and improving performance of the signaling systems. The other is to utilize the existing infrastructure more efficiently. It is generally believed that the railway operation work can be divided into three levels, strategic level, tactical level, and operational level [1]. The strategic level is about transportation pattern selecting, which is related to the national transportation policy. And the middle one, tactical level, is on the line plan designing, which is also called service plan, determining the trains number, paths, stops, and so forth.

And the line plan is divided into several parts, which are the origin and destination stations determining, the trains number calculation, the train pathing, and the stops setting. Among them, train pathing is the most important step to design the whole line plan, which is the basis of stops setting. Generally, the paths of the trains are relatively steady, according to the yearly railway line plan. However, there are occasional railway accidents which reduce the capability of the railway line and make it impossible for the trains to run on the planned paths. It is necessary to find the substitute path for the trains. On the other hand, with the increase of the available rail, the topology structure of the railway network is changing profoundly. A new railway network is forming gradually, which makes it possible that more than one path can be found for the trains and train trips can be allocated on the paths.

The organization of this paper is as follows. Following this introduction, we first discuss the related works on the problem in Section 2. Then we build the train pathing model based on fuzzy linear programming in Section 3. In Section 4, we analyze the fuzzy coefficients in the train pathing model and design a new algorithm to solve the fuzzy linear model. Furthermore, we study the values range of the fuzzy coefficients, designing a method to describe the uncertainty of the fuzzy characters of the coefficients. We prove the availability of the model and the efficiency of the algorithm with a computation case in Section 5. In Section 6, we draw a conclusion.

## 2. Related Works

Caprara et al. [2] and D'Ariano and Pranzo [3] grouped the major published railway operation as line planning, timetabling, platforming, rolling stock management, shunting, and crew planning. Train pathing is a key step of line planning, which belongs to the tactical level. Train timetables are usually specified after the train pathing [4]. So it is a must to determine the path plan before timetabling, especially in emergencies.

There are two kinds of approaches to solve the train pathing problem in the limited number of publications, the mathematical approaches and heuristic approaches.

Carey [5] presented a mathematical model, algorithms, and strategy for pathing trains of different speeds and stopping patterns for a double track rail line dedicated to trains in one direction. The model included track assignment to trains within stations (choice of platform) and between stations (choice among multiple lines). Station layout was also considered in the model. He applied the model to a small network and found acceptable solution times. He further extended the model from one-way to two-way tracks [6]. Carey and Lockwood [7] developed a model and algorithm for the TPP for one train line with station stops and solved instances of 10 trains and 10 links. All the trains on the line travel in the same direction. D'Ariano et al. [8] hired a branch-and-bound algorithm for sequencing train movements, while a local search algorithm is developed for rerouting optimization purposes. And they analyzed different types of disturbances, including train delays and blocked tracks. The authors of this paper defined generating paths in emergencies as a *k*-shortest path problem and proposed the method to solve it, innovating* Dijkstra* algorithm [9]. Fuzzy programming is introduced to solve the train routing and pathing problem recently. And Yang et al. [10] considered the fuzziness in the railway transportation system and proposed a min-max chance-constrained programming model to solve the freight train routing problem with fuzzy information.

Heuristic is also hired in train pathing problem solving in recent years. Carey and Crawford [11] developed a heuristic to solve for a plan that brings trains through a rail corridor with multiple lines and multiple stations. They started from algorithms that schedule trains at a single train station and extend these to handle a series of complex stations linked by multiple one-way lines in each direction, traversed by trains of differing types and speeds. The algorithm was based on a set of rules to resolve the conflicts. Lee and Chen [12] also presented a heuristic that includes both train pathing and train timetabling and has the ability to solve real-sized instances. This heuristic allowed the operation time of trains to depend on the assigned track. Blum and Eskandarian [13] used a delegation model to improve agent collaboration as an effective way to improve the efficiency of an A-Team for railroad flow optimization, including train pathing and railroad routing. Erlebach et al. [14] studied the method to assign trains to satisfy scheduled routes in a cost efficient way and proposed approximation algorithms. Törnquist [15] presented a heuristic approach for railway traffic rescheduling during disturbances and a performance evaluation for various disturbance settings using data for a large part of the Swedish railway network. Dorfman and Medanic [16] developed a local feedback-based travel advance strategy, using a discrete event model of train advances along lines of the railway to quickly handle perturbations on the railway network, including train pathing. Caimi et al. [17] addressed the problem of generating conflict-free train schedules on a microscopic model of the railway infrastructure and developed an alternative model using the sequence of resources that each train path passes, encoded in a resource tree. They showed that the number of maximal conflict cliques is linear in the number of train paths and verified the model with real-world data from the Swiss Federal Railways. Lusby et al. [18] described a set packing inspired formulation of train routing problem and developed a branch-and-price based solution approach. They verified the model with the test instance arising in Germany and supplied by the major German railway company, Deutsche Bahn. Pellegrini et al. [19] proposed a mixed-integer linear programming formulation for tackling this problem, representing the infrastructure with fine granularity. They tackled randomly generated instances representing traffic in the control area named triangle of Gagny and instances obtained from the real timetable of the control area including the Lille-Flandres station (both in France) and found that negative impact of a rough granularity on the delay suffered by trains was remarkable and statistically significant. Li et al. [20] constructed a train routing model combined with a train scheduling problem, which is a 0-1 mixed-integer nonlinear programming problem. They designed a tabu search procedure to further improve the route schemes. Train repathing problem is similar to the train routing problem in several aspects. So their approach also gave us some enlightenment.

All these related works gave us much enlightenment when we built the train repathing model and designed the algorithm to solve it. However, the fuzzy characteristics in train repathing problem were not considered in these publications, and the rail segments capability is not set to be the restriction when building the model in most of the publications. So we also focus on the processing of fuzzy coefficients processing in the train repathing model.

## 3. Train Repathing Model

The objective is to reduce the total cost as much as possible. The input data include the paths between two stations, the capability of the rail segments affected and the stations affected, and all the trains information needing changing paths.

### 3.1. Basic Assumption

1. Assumption on crew. We took it for granted that the crew resource is enough to cope with the trains flow distribution.
2. Assumption on rails availability. We took it for granted that all the trains can run on all the types of rails.

### 3.2. Graph Based Description of Rail Networks

*G* = (*V*, *E*) is a railway network that is constructed of all kinds of rails. *V* is the set of vertexes in the railway network. *E* is the set of edges in the railway network. *V* includes the stations of the existent normal speed railway, the existent intercity railway, and the newly built railway. And *E* not only includes the rail segments of the different types of railway, but also includes the links between different types of rails.

### 3.3. Available Paths Set Generating

According to the method in our previous research paper [9], we can generate the paths set *P* when an emergency occurs. The calculating steps are as follows.

**Step 1 .**

To find the shortest path with* Dijkstra* algorithm between the origin and the destination and put the shortest path, length of the shortest paths, and nodes on the shortest path into the path array *P*, distance array *D*, and node array *M*.

**Step 2 .**

To find neighbor nodes of the shortest path in array *P* and put them into another array *N*.

**Step 3 .**

To calculate the distance of *n*-shortest path of *v*_*s*_-*v*_*t*_-*v*_*j*_-*v*_*e*_, which pass through neighbor *v*_*t*_ and put it into array *T*. *v*_*j*_ is a node on *n*-shortest path.

**Step 4 .**

To order the lengths values in array *T*. To select the smallest one and put the relative path in array *P*. To add 1 to the number of the shortest paths.

**Step 5 .**

If the total capability of the all the shortest paths reaches to the required capability, stop the calculation. Else, go Step 2.

Then we can generate a set of shortest paths for the train operation and the sum of capabilities of all the paths in the path set is enough for train repathing work.

### 3.4. Optimization Objectives

The cost can be divided into three parts, the running cost, transferring cost, and social effect punishment cost. The running cost is an inevitable cost, which occurs during the running process.

When distributing the trains on paths, which consist of different kinds of rails, the transferring cost and the social effect punishment cost occur. In this paper, transferring cost is used to denote the cost occurrence when a train transfers from one type of rail line to another type of rail line.* Transferring cost* includes equipment cost, technology operation cost, and abrasion cost [21]. Among them the equipment costs and abrasion costs are very difficult to calculate accurately. The technology operation cost is related to profit of the railway bureau and the technology operation quantity. The* transferring cost* also depends on the rail grade, train type, and the fact whether a ferry-locomotive is needed, which is very difficult to calculate exactly. But we can set the value range of it.

The social effect punishment cost is related to the passenger satisfaction, which is also difficult to figure out and the value range can be defined.

The* transferring cost* and social effect punishment cost are more characterized by fuzziness in actual transportation operation, especially in emergencies. The coefficient can be expressed by some fuzzy functions, such as triangular fuzzy function and trapezoidal fuzzy function. All the optimization objectives can be compromised to some extent. As long as the values of the optimization objectives reach into a certain value range, it is considered that the optimization process is successful. We designed the method to cope with the fuzzy character of all the objectives and the algorithm to solve the trains flow distribution problem.

### 3.5. Train Distribution Model

The decision variables and parameters are as follows:

 
*V*: the set of all the station nodes in the network considered in this paper,
 
*k*: index of train type,
 
*M*: the number of the train types,
 
*p*: index of path,
 
*P*: the set of available *p*,
 
*Q* ^*p*^: the set of all segments on path *p*,
 
*R* ^*p*^: the set of all stations on path *p*,
 
*s*: index of station,
 
*t*: index of station,
 
*v*_*s*_, *v*_*t*_: station node *s* and station node *t*,
 
*e*_*t*_^*s*^: the segment from station *v*_*s*_ to station *v*_*t*_,
 
*d*_*t*_^*s*^: the length from station *v*_*s*_ to station *v*_*t*_,
 
*n*_*p*_^*k*^: the number of the *k* type trains allocated at path *p*,
 
*δ*_*t*_^*s*^: the transferring cost coefficient from station *v*_*s*_ to station *v*_*t*_. Station *s* and station *t* are two stations from different types of rails,
 
*ξ*_*t*_^*s*^: the running cost coefficient of segment from station *v*_*s*_ to station *v*_*t*_. Station *s* and station *t* are the two stations from the same types of rails,
 
*λ*_*p*_^*k*^: the social cost punishment coefficient if a *k* type train was allocated at path *p*. Station *s* and station *t* are the two stations from different types of rails,
 
*D*_*t*_^*s*^: the capability of segment *e*_*t*_^*s*^,
 
*B*_*s*_: the capability of station *s*,
 
*N*+: the set of positive integers.

#### 3.5.1. Formulation of Objectives

The costs are listed and analyzed in Section 3.4. Here we formulate the costs, respectively.

(1) *Transferring cost*:
  (1) $$\begin{array}{c} Z_{T}=\overset{M}{\underset{k=1}{\sum }}\overset{}{\underset{p\in P}{\sum }}\underset{e_{t}^{s}\in Q^{p}}{\sum }\delta_{t}^{s}n_{p}^{k}. \end{array}$$
(2) Running cost:
  (2) $$\begin{array}{c} Z_{R}=\overset{M}{\underset{k=1}{\sum }}\underset{p\in P}{\sum }n_{p}^{k}\left(\underset{e_{t}^{s}\in Q^{p}}{\sum }\xi_{t}^{s}d_{t}^{s}\right). \end{array}$$
(3) Social effect punishment cost:
  (3) $$\begin{array}{c} Z_{S}=\overset{M}{\underset{k=1}{\sum }}\overset{}{\underset{p\in P}{\sum }}\lambda_{p}^{k}n_{p}^{k}. \end{array}$$

Then, we normalized the three kinds of cost by adding the coefficients, *δ*_*t*_^*s*^, *ξ*_*t*_^*k*^, and *λ*_*p*_^*k*^. Then the total cost of the model is as follows:

(4) $$\begin{array}{c} \min Z_{TT}=Z_{T}+Z_{R}+Z_{S}. \end{array}$$

It is equal to

(5) min⁡ZTT=∑k=1M∑p∈P∑ets∈Qpδtsnpk+∑k=1M∑p∈Pnpk(∑ets∈Qpξtsdts) +∑k=1M∑p∈Pλpknpk.

#### 3.5.2. Constraints of the Model

There are many constraints when assigning all the trains to the available paths. The main constraints to be considered are as follows.

(1) Segments capacity constraints. The number of trains running through segment *e*_*t*_^*s*^ cannot surpass its capability. Consider
  (6) $$\begin{array}{c} \overset{M}{\underset{k=1}{\sum }}\underset{p\in P}{\sum }\overset{}{\underset{e_{t}^{s}\in Q^{p}}{\sum }}n_{p}^{k}\leq D_{t}^{s} \end{array}$$
  *D*_*t*_^*s*^ is the capability of segment *e*_*t*_^*s*^.
(2) Stations capacity constraints. The capability of every station in the railway network is bigger than the number of all the trains inbound and outbound. Consider
  (7) $$\begin{array}{c} \overset{M}{\underset{k=1}{\sum }}\underset{p\in P}{\sum }\overset{}{\underset{v_{s}^{}\in R^{p}}{\sum }}n_{p}^{k}\leq B_{s}^{}. \end{array}$$
(3) Nonnegativity constraints:
  (8) $$\begin{array}{c} n_{p}^{k}\in N+\;\text{or}\;\;n_{p}^{k}=0. \end{array}$$

We can see that the model is a linear integer programming model.

## 4. Fuzzy Coefficients Processing and Train Repathing Model Solution

There are numerous fuzzy numbers in the model built up in Section 3.5. So we first present the method to process fuzzy numbers of the model. Then based on the processing, we propose the steps to set the model with optimization software LINGO 11.0.

### 4.1. Fuzzy Coefficients Processing

A fuzzy number is a generalization of a regular, real number in the sense that it does not refer to one single value but rather to a connected set of possible values, where each possible value has its own weight between 0 and 1. This weight is called the membership function. In the engineering computation field, many elements cannot be described with definite numbers, while we can tell how much they belong to a certain range. The degree can be represented by fuzzy numbers. It is a powerful tool to describe this kind of element.

Generally, fuzzy linear programming models can be divided into three groups. The first group of models has fuzzy resources in the constraints of the model. That is to say, the resources of the constraints are fuzzy which should be described with the fuzzy membership functions. The second group of models has the fuzzy coefficients of the objectives. The fuzzy numbers occur in the optimization goal equations. The last group has the characteristics of the above two groups. They both have the fuzzy resources constraints and the fuzzy objective coefficients.

In this paper, there are several objective coefficients which are uncertain and difficult to obtain and we model the problem as the second group. Transferring cost is a typical fuzzy number and it is very difficult to get. When disturbances occur, the price assessment of transferring cost is with more fuzziness. Fuzzy factors could be defined with fuzzy numbers. Typical fuzzy membership functions are triangular function, trapezoid function, and so on. When the fuzzy degree is out of control with the typical definition of the fuzzy factors, we should improve the function to deal with the situation.

It is clear that the train distribution model is a fuzzy linear integer programming model. The tolerance method is the most typical method. In this section, we introduce the tolerance method and present a new method to solve the fuzzy linear integer programming model. And we propose a method to enlarge the fuzzy coefficients support.

In some occasions, the boundaries of the value range are also difficult to determine, especially in emergencies. So we design a method, hiring a function *F*(*x*) to expand the value range.

**Figure 1:**
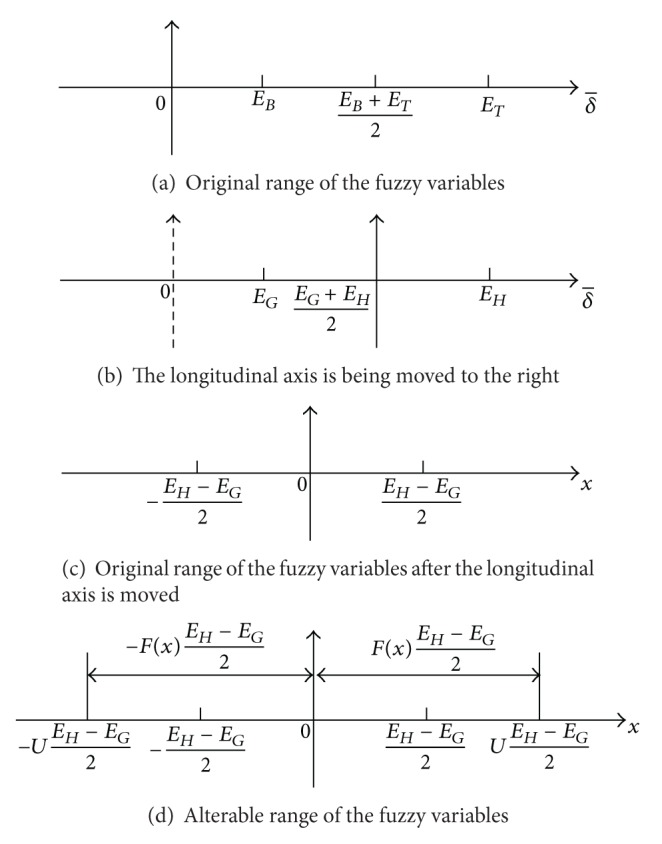
Design of alterable range of the fuzzy variables.

Set *E*_*H*_ to be the optimistic value of *δ* and *E*_*G*_ to be the pessimistic value. Then *E*_*G*_ ≤ *δ* ≤ *E*_*H*_, *E*_*G*_ ≥ 0, *E*_*H*_ ≥ 0, and $\overset{-}{\delta }$ is the average value of *δ*, as shown in Figure 1(a).

Set

(9) $$\begin{array}{c} x=\delta -\frac{E_{G}+E_{H}}{2}. \end{array}$$

The change is shown in Figure 1(b).

Then

(10) $$\begin{array}{c} -\frac{E_{H}-E_{G}}{2}\leq x\leq \frac{E_{H}-E_{G}}{2}. \end{array}$$

So *x* is symmetrical by *y* axis, as shown in Figure 1(c).

Then, *F*(*x*) is hired to expand the value range of *x*. Consider

(11) $$\begin{array}{c} -F\left(x\right)\frac{E_{H}-E_{G}}{2}\leq x\leq F\left(x\right)\frac{E_{H}-E_{G}}{2}. \end{array}$$

So

(12) −F(x)EH−EG2+EG+EH2 ≤δ≤F(x)EH−EG2+EG+EH2.

It can be seen in Figure 1(d).

That is to say,

(13) −F(δ−EG+EH2)EH−EG2+EG+EH2 ≤δ≤F(δ−EG+EH2)EH−EG2+EG+EH2.

So the fuzzy coefficients value range is as follows after steps above:

(14) [−F(δ−EG+EH2)EH−EG2+EG+EH2, F(δ−EG+EH2)EH−EG2+EG+EH2].

It can be seen that the expanded value range is related to the original range and the average value of the fuzzy coefficients. This method can deal with the fuzzy coefficients flexibly, making the coefficients close to the real cost as much as possible.

### 4.2. Steps to Solve Train Distribution Model

It is obvious that the programming model is a fuzzy linear programming with fuzzy objective coefficients. Since some coefficients of the objective are fuzzy, we must deal with them first. We design the method to express the coefficients with the pessimistic value, average value, and optimistic value. Since *E*_*H*_ is the optimistic value of *δ* and *E*_*G*_ is the pessimistic value of *δ*, we set *E*_*A*_ to be the average value of *δ*. We assume that *δ* = *w*_1_ *E*_*H*_ + *w*_2_ *E*_*G*_ + (1 − *w*_1_ − *w*_2_)*E*_*A*_, where *w*_1_ and *w*_2_ are the weights of the optimistic value and pessimistic value, respectively. We can see that the fuzzy linear programming can be transferred into different deterministic linear programming with different pairs of *w*_1_ and *w*_2_. Then the steps to solve the problem are as follows.

**Step 1 .**

Set *w*_2_ = 0.1.

**Step 2 .**

Set  *w*_2_ = *w*_2_ + 0.1.

**Step 3 .**

Set *w*_1_ to be 0.1; then solve the linear programming with LINGO 11.0.

**Step 4 .**

Repeat Step 2 with *w*_1_ increasing 0.1 a time.

**Step 5 .**

Record the value of *w*_1_, *w*_2_, and the computing results.

**Step 6 .**

Go to Step 2 and repeat the process until *w*_2_ = 1.5.

**Step 7 .**

Select the satisfying solution for the model.

## 5. Case Study

### 5.1. Case Scenario

**Figure 2:**
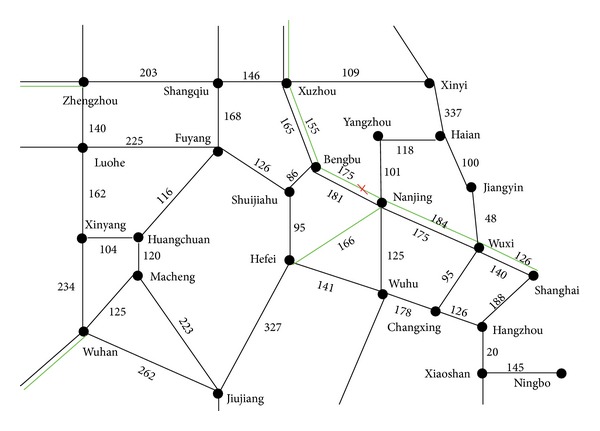
Railway network around the emergency place. Note: the green lines stand for high speed rails and the black lines stand for low speed rails.

It is assumed that there is an emergency at DK856 + 321 on the Beijing-Shanghai high speed railway. Then the trains cannot run through the segment of East Xuzhou to Bengbu. And the time required to recover is 4 to 8 hours. The railway network around the emergency place is shown in Figure 2. We will study the trains flow distribution problem on the down-going direction.

### 5.2. Trains to Be Repathing

Trains arriving at Xuzhou joint from 8 to 12 are as follows.

1. The high speed trains (shorthand: H): G301, G303, G305, G101, G103, G105, G107, G109, G111, and G113.
2. Multiple units (shorthand: M): D88/5.
3. T trains and K trains (shorthand: T&M): K58/5, K518/5, and K101/4/1.
4. Normal speed trains (shorthand: N): 1230/27.
5. Low speed trains (shorthand: L): 10135, 10625, 11301, 23005, and 11305.
6. Temporary trains (shorthand: T): none.
7. Other trains (shorthand: O): none.

### 5.3. Available Paths

**Figure 3:**
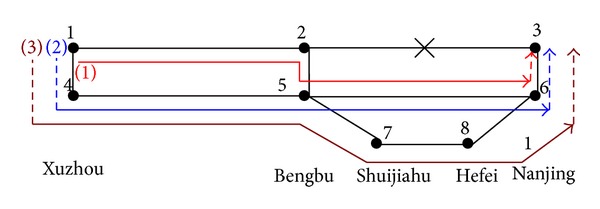
Paths between Xuzhou and Nanjing in an emergency.

According to the method in our previous paper [9], we generate the available paths according to the succinct description in Section 3.3, shown in Table 1 and Figure 3.

And lengths of every segments on the paths are shown in Table 2.

### 5.4. Distribution Plan

#### 5.4.1. Specification of the Train Distribution Model

We can see that there are three available paths in the partial railway network, which are marked (1), (2), and (3), as shown in Figure 3. Eight stations and six segments are in the network.

Now the goal is to allocate the trains on the three paths in Table 1.

We specified the model as follows:

(15) min⁡ Z=δ41(n21+n31)+δ52(n11)+δ36(n11+n21+n31)+ξ21n11×155+ξ54(n21+n22+n23+n24+n25+n31+n32+n33+n34+n35)×165+ξ65(n11+n21+n22+n23+n24+n25)×181+ξ75(n31+n32+n33+n34+n35)×86+ξ87(n31+n32+n33+n34+n35)×95+ξ68(n31+n32+n33+n34+n35)×166+λ11(n11)+λ21(n21)+λ31(n31)+λ32n32+λ33n33+λ34n34+λ35n35s.t. n11≤C21n21+n31+n22+n32+n23+n33+n24+n34+n25+n35≤C54n11+n21+n22+n23+n24+n25≤C65n31+n32+n33+n34+n35≤C75n31+n32+n33+n34+n35≤C87n31+n32+n33+n34+n35≤C68n11≤B2n21+n31+n22+n32+n23+n33+n24+n34+n25+n35≤B4n11+n21+n31+n22+n32+n23+n33+n24+n34+n25+n35≤B5n11+n21+n31+n22+n32+n23+n33+n24+n34+n25+n35≤B6n31+n32+n33+n34+n35≤B7n31+n32+n33+n34+n35≤B8n11+n21+n31=10n12+n22+n32=1n13+n23+n33=3n14+n24+n34=1n15+n25+n35=5npk=0 or npk∈N+, k=1,2,3,4,5;p=1,2,3.

*δ*_4_^1^, *δ*_5_^2^, and *δ*_3_^6^ are got from the publication [21].

*λ*_3_^2^, *λ*_3_^3^, *λ*_3_^4^, and *λ*_3_^5^ are social effect punishment cost coefficient when the 2th, 3th, 4th, and 5th kinds of trains are allocated on path 3. They can be attained by the Delphi method.

We got the train running cost coefficients according to the data listed in two publications [22, 23]. The coefficients are as follows:

(16) $$\begin{array}{c} \xi_{2}^{1}=\xi_{6}^{8}=325,\;\;\xi_{5}^{4}=\xi_{6}^{5}=\xi_{7}^{5}=\xi_{8}^{7}=92.4. \end{array}$$

The capabilities of the related segments and stations are as follows:

(17) $$\begin{array}{c} C_{2}^{1}=115,\;\;C_{5}^{4}=20,\;\;C_{6}^{5}=20,\\ C_{7}^{5}=12,\;\;C_{8}^{7}=12,\;\;C_{6}^{8}=62,\\ B_{2}=30,\;\;B_{4}=32,\;\;B_{5}=38,\\ B_{6}=30,\;\;B_{7}=20,\;\;E_{G}B_{8}=70. \end{array}$$

Set *δ*_4_^1^ ~ (2800,3000,3200), *δ*_5_^2^ ~ (2600,2800,3000), *δ*_3_^6^ ~ (3000,3200,3400), *λ*_1_^1^ ~ (20000,22000,24000), *λ*_2_^1^ ~ (60000,64000,68000), *λ*_3_^1^ ~ (80000,86000,92000), and *λ*_3_^2^, *λ*_3_^3^, *λ*_3_^4^, and *λ*_3_^5^ ~ (10000,11000,12000).

For example, *δ*_4_^1^ ~ (2800,3000,3200) means that the largest value of the fuzzy number *δ*_4_^1^ is 3200 and the smallest value is 2800. The average value is 3000. That is to say, that *E*_*H*_ = 3200 and *E*_*G*_ = 2800.

If at this time *w*_1_ = 0.1 and *w*_2_ = 0.5, *δ*_4_^1^ = 0.1∗3200 + 0.5∗2800 + (1 − 0.1–0.5)∗3000 = 2920. All the other fuzzy coefficients can be calculated out in the same way. The fuzzy linear programming model is turned into a deterministic linear programming model, which can be easily solved with the software LINGO 11.0.

It should be noticed that there is little* transferring cost* at Hefei and Nanjing on path (3), for the segment between Hefei and Nanjing is a high speed segment. But the transferring operation is in the station, and the cost is very little. So this* transferring cost* is not taken into consideration in this model.

#### 5.4.2. Solutions

(1) Solving the problem in original value range.

We compute the results, respectively, while *w*_1_ is assigned to be 0.1 to 1.5. The results are shown in Table 3(a).

It can be seen that the fuzzy coefficients are bigger than the average value. It means that the smaller the values of the fuzzy coefficients are, the bigger the objective value is. It is obvious that the relative results are not satisfying. So the results in shadowed part in Tables 3(a) and 3(b) are the unreasonable solution.

When *w*_1_ is 0.5, the fuzzy coefficients are equal to their average value. This is the most possible situation of the reality, in which the fuzzy membership is 1. The total cost is 1.2778E06 at this point, which is the highest.

When *w*_1_ is set to be 0.7 to 1.5, solution of the problem is not changed. That is to say, all the solutions with the membership under 0.8 are the same. However, the objective value changes with the fuzzy coefficients changing. According to the rule that the solution with maximal membership value and the minimal objective value should be selected, the solution is taken as *n*_1_^1^ = 10, *n*_2_^1^ = 0, *n*_2_^2^ = 1, *n*_2_^3^ = 3, *n*_2_^4^ = 1, *n*_2_^5^ = 5, and *n*_3_^1^ = *n*_3_^2^ = *n*_3_^3^ = *n*_3_^4^ = *n*_3_^5^ = 0. The objective value is 1.2659E06.

(2) Solving the problem in variable value range.

Set the variable function *F*(*x*) = 2. So the value range is twice as large as the original value range. That is to say, the expanding coefficient is 2. We take the calculation of *δ*_4_^1^, for instance. Since *F*(*x*) = 2, *E*_*G*_ = 2800, and *E*_*H*_ = 3200, the value range of *δ*_4_^1^ is

(18) [−F(x)EH−EG2+EG+EH2,F(x)EH−EG2+EG+EH2] =[−2×3200−28002+3200+28002,   2×3200−28002+3200+28002] =[2600,3400].

All the other coefficients value range can be obtained by the same means. In fact, we expand the value range by this means based on the original value range, according to (12).

The expanded value ranges are as follows:

*δ*_4_^1^ ~ (2600,3000,3400), *δ*_5_^2^ ~ (2400,2800,3200), *δ*_3_^6^ ~ (2800,3200,3600), *λ*_1_^1^ ~ (18000,22000,26000), *λ*_2_^1^ ~ (58000,64000,70000), *λ*_3_^1^ ~ (78000,86000,94000), and *λ*_3_^2^, *λ*_3_^3^, *λ*_3_^4^, and *λ*_3_^5^ ~ (8000,11000,14000).

The computation results are shown in Table 3(b).

**Figure 4:**
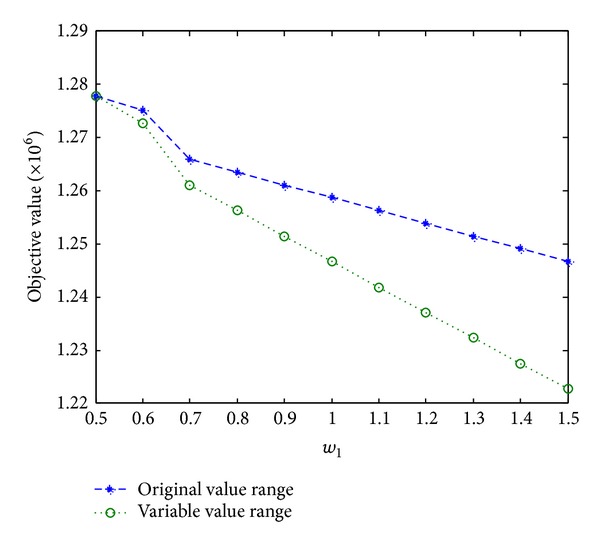
Solution of the train paths distributing model.

From Table 3(b) we can see that the objective value is reduced when the membership values are the same, as shown in Figure 4.

The difference between the objective value calculated with the original value range and that calculated with the variable value range becomes obvious from the point where *w*_1_ is 0.7. And the difference is becoming more and more obvious till the point where *w*_1_ is 1.5. The objective value is 1.2467E06 with the original value range and the objective value is 1.2227E06 with the variable value range. So we can see that the objective value is optimized when we make the value range variable.

The solution with the variable value range is also *n*_1_^1^ = 10, *n*_2_^1^ = 0, *n*_2_^2^ = 1, *n*_2_^3^ = 3, *n*_2_^4^ = 1, *n*_2_^5^ = 5, *n*_3_^1^ = *n*_3_^2^ = *n*_3_^3^ = *n*_3_^4^ = *n*_3_^5^ = 0.

**Figure 5:**
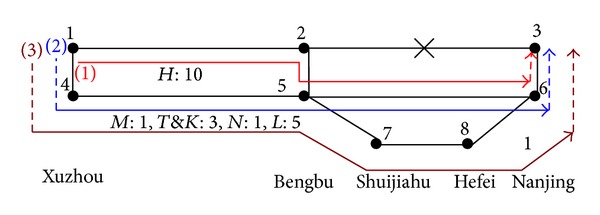
Solution of the train paths distributing model shown on the railway network graph.

And we can see in Figure 5 that all the trains are allocated on path 1 and path 2. No train is allocated on path 3. It is related to the required capacity which is 40, when searching for the available paths. However, the number of trains needing to be allocated is 20. It is necessary to set the required capacity to be bigger than the number of trains needing to be allocated. For one thing, the accurate number of the trains needing to be allocated is difficult to forecast. For another, it is a must to reserve extra capacity to deal with the uncertain situation of the reality.

The methods presented in this paper can give the optimized solution, satisfying the fuzzy membership constraint. So we can deal with the fuzzy character of the trains flow distribution model to approach the reality as best as we can. We can propose several available solutions, at different fuzzy membership level for the managers to make the decision.

## 6. Conclusion

This paper proposes a feasible, effective approach to solve the fuzzy programming problems in railway transportation. We first present a model for distributing trains on paths, offering the theory basis for train dispatching on China railway network. Then we integrate the* transferring cost*, running cost, and social effect punishment cost to design the objective of the train distribution model. The character of the coefficient of the costs is described with fuzzy membership function. And we present a method to expand the fuzzy number value range, supporting the algorithm to solve the model. A triangular membership function is designed to turn the fuzzy programming model into definitive programming problem. And the detailed steps to solve the model are given.

The method presented can also be used to solve other problems in railway transportation organization. We can deal with the fuzzy character of the passenger transportation and freight transportation requirement in service planning. It also may work in fuzzy objectives in the Electric Multiple Units timetable designing, the work time in crew schedule designing. And in solving the routing problem of trains at stations, we can also hire the method to describe the fuzzy character when the operation time has the fuzzy characters.

## Figures and Tables

**Table 1:** Available paths from Xuzhou to Nanjing (by *C*-enough plan).

Number	Available paths	Length (km)
1	1-2-5-6-3	336
2	1-4-5-6-3	346
3	1-4-5-7-8-6-3	502

**Table 2:** Lengths of segments in the paths.

Segment number	Segments	Length (km)
1	1-2	155
2	4-5	165
3	5-6	181
4	5-7	86
5	7-8	95
6	8-6	166

**Table:** (a) In original value range

*w*_1_	*F*1	*F*2	*F*3	*F*4	*F*5	*F*6	*F*7	*n*_1_^1^	*n*_2_^1^	*n*_3_^1^	*n*_2_^2^	*n*_3_^2^	*n*_2_^3^	*n*_3_^3^	*n*_2_^4^	*n*_3_^4^	*n*_2_^5^	*n*_3_^5^	*S*_mf_	*Z*(10^∧^6)
**0.1**	**3080**	**2880**	**3280**	**22800**	**65600**	**88400**	**11400**	**9**	**1**	**0**	**1**	**0**	**2**	**1**	**1**	**0**	**3**	**2**	**4.20**	**1.3684**
**0.2**	**3060**	**2860**	**3260**	**22600**	**65200**	**87800**	**11300**	**9**	**1**	**0**	**1**	**0**	**2**	**1**	**1**	**0**	**4**	**1**	**4.90**	**1.3389**
**0.3**	**3040**	**2840**	**3240**	**22400**	**64800**	**87200**	**11200**	**9**	**1**	**0**	**1**	**0**	**2**	**1**	**1**	**0**	**4**	**1**	**5.60**	**1.3361**
**0.4**	**3020**	**2820**	**3220**	**22200**	**64400**	**86600**	**11100**	**9**	**1**	**0**	**1**	**0**	**3**	**0**	**1**	**0**	**4**	**1**	**6.30**	**1.3068**
0.5	3000	2800	3200	22000	64000	86000	11000	9	1	0	1	0	3	0	1	0	5	0	7.00	1.2778
0.6	2980	2780	3180	21800	63600	85400	10900	9	1	0	1	0	3	0	1	0	5	0	6.30	1.2752
0.7	2960	2760	3160	21600	63200	84800	10800	10	0	0	1	0	3	0	1	0	5	0	5.60	1.2659
0.8	2940	2740	3140	21400	62800	84200	10700	10	0	0	1	0	3	0	1	0	5	0	4.90	1.2635
0.9	2920	2720	3120	21200	62400	83600	10600	10	0	0	1	0	3	0	1	0	5	0	4.20	1.2611
1.0	2900	2700	3100	21000	62000	83000	10500	10	0	0	1	0	3	0	1	0	5	0	3.50	1.2587
1.1	2880	2680	3080	20800	61600	82400	10400	10	0	0	1	0	3	0	1	0	5	0	2.80	1.2563
1.2	2860	2660	3060	20600	61200	81800	10300	10	0	0	1	0	3	0	1	0	5	0	2.10	1.2539
1.3	2840	2640	3040	20400	60800	81200	10200	10	0	0	1	0	3	0	1	0	5	0	1.60	1.2515
1.4	2820	2620	3020	20200	60400	80600	10100	10	0	0	1	0	3	0	1	0	5	0	0.70	1.2491
1.5	2800	2600	3000	20000	60000	80000	10000	10	0	0	1	0	3	0	1	0	5	0	0.00	1.2467

**Table:** (b) In variable value range

*w*_1_	*F*1	*F*2	*F*3	*F*4	*F*5	*F*6	*F*7	*n*_1_^1^	*n*_2_^1^	*n*_3_^1^	*n*_2_^2^	*n*_3_^2^	*n*_2_^3^	*n*_3_^3^	*n*_2_^4^	*n*_3_^4^	*n*_2_^5^	*n*_3_^5^	*S*_mf_	*Z*(10^∧^6)
**0.1**	**3160**	**2960**	**3360**	**23600**	**66400**	**89200**	**12200**	**9**	**1**	**0**	**1**	**0**	**2**	**1**	**1**	**0**	**3**	**2**	**4.20**	**1.3804**
**0.2**	**3120**	**2920**	**3320**	**23200**	**65800**	**88400**	**11900**	**9**	**1**	**0**	**1**	**0**	**2**	**1**	**1**	**0**	**4**	**1**	**4.90**	**1.3473**
**0.3**	**3080**	**2880**	**3280**	**22800**	**65200**	**87600**	**11600**	**9**	**1**	**0**	**1**	**0**	**2**	**1**	**1**	**0**	**4**	**1**	**5.60**	**1.3412**
**0.4**	**3040**	**2840**	**3240**	**22400**	**64600**	**86800**	**11300**	**9**	**1**	**0**	**1**	**0**	**3**	**0**	**1**	**0**	**4**	**1**	**6.30**	**1.3094**
0.5	3000	2800	3200	22000	64000	86000	11000	9	1	0	1	0	3	0	1	0	5	0	7.00	1.2778
0.6	2960	2760	3160	21600	63400	85200	10700	9	1	0	1	0	3	0	1	0	5	0	6.30	1.2728
0.7	2920	2720	3120	21200	62800	84400	10400	10	0	0	1	0	3	0	1	0	5	0	5.60	1.2611
0.8	2880	2680	3080	20800	62200	83600	10100	10	0	0	1	0	3	0	1	0	5	0	4.90	1.2563
0.9	2840	2640	3040	20400	61600	82800	9800	10	0	0	1	0	3	0	1	0	5	0	4.20	1.2515
1.0	2800	2600	3000	20000	61000	82000	9500	10	0	0	1	0	3	0	1	0	5	0	3.50	1.2467
1.1	2760	2560	2960	19600	60400	81200	9200	10	0	0	1	0	3	0	1	0	5	0	2.80	1.2419
1.2	2720	2520	2920	19200	59800	80400	8900	10	0	0	1	0	3	0	1	0	5	0	2.10	1.2371
1.3	2680	2480	2880	18800	59200	79600	8600	10	0	0	1	0	3	0	1	0	5	0	1.60	1.2323
1.4	2640	2440	2840	18400	58600	78800	8300	10	0	0	1	0	3	0	1	0	5	0	0.70	1.2275
1.5	2600	2400	2800	18000	58000	78000	8000	10	0	0	1	0	3	0	1	0	5	0	0.00	1.2227
